# Renal Replacement Therapy

**DOI:** 10.12688/f1000research.6935.1

**Published:** 2016-01-25

**Authors:** Zaccaria Ricci, Stefano Romagnoli, Claudio Ronco

**Affiliations:** 1 Department of Cardiology and Cardiac Surgery, Pediatric Cardiac Intensive Care Unit, Bambino Gesù Children's Hospital, Rome, Italy; 2 Department of Health Science, Section of Anesthesiology and Intensive Care, University of Florence, Florence, Italy; 3 Department of Anesthesia and Intensive Care, Azienda Ospedaliero Careggi, Florence, Italy; 4 Department of Nephrology, Dialysis and Transplantation, San Bortolo Hospital, Vicenza, Italy; 5 International Renal Research Institute, San Bortolo Hospital, Vicenza, Italy

**Keywords:** Renal Replacement Therapy, acute kidney injury, dialysis, anticoagulation strategies, blood purification, critical care nephrology

## Abstract

During the last few years, due to medical and surgical evolution, patients with increasingly severe diseases causing multiorgan dysfunction are frequently admitted to intensive care units. Therapeutic options, when organ failure occurs, are frequently nonspecific and mostly directed towards supporting vital function. In these scenarios, the kidneys are almost always involved and, therefore, renal replacement therapies have become a common routine practice in critically ill patients with acute kidney injury. Recent technological improvement has led to the production of safe, versatile and efficient dialysis machines. In addition, emerging evidence may allow better individualization of treatment with tailored prescription depending on the patients’ clinical picture (e.g. sepsis, fluid overload, pediatric). The aim of the present review is to give a general overview of current practice in renal replacement therapies for critically ill patients. The main clinical aspects, including dose prescription, modality of dialysis delivery, anticoagulation strategies and timing will be addressed. In addition, some technical issues on physical principles governing blood purification, filters characteristics, and vascular access, will be covered. Finally, a section on current standard nomenclature of renal replacement therapy is devoted to clarify the “Tower of Babel” of critical care nephrology.

## Introduction

Critically ill patients with severe diseases and multisystem organ failure are currently frequently admitted to and treated in the intensive care unit (ICU). Although the identification and management of multisystem organ failure has improved, the incidence has increased over the last half-century ^1^. Therapeutic options in the setting of multisystem organ failure are mostly aimed at supporting vital functions. The kidneys are almost always involved in such a syndrome, and dialytic techniques are routinely used in the ICUs to treat severe acute kidney injury (AKI) ^2^.

Current practice in renal replacement therapy (RRT) for adult critically ill patients, with specific details on technical features and clinical applications, will be reviewed.

## Technical issues

### The technological evolution of RRT

Peter Kramer in 1977 described the first continuous form of dialysis specifically dedicated to critically ill patients: continuous arterio-venous hemofiltration (CAVH) ^3^. In CAVH, blood flow in the circuit was driven by a spontaneous arterio-venous pressure gradient and spontaneous ultrafiltration (UF) occurred depending on the transmembrane pressure (TMP) gradient. The arterio-venous pressure gradient was dependent on the mean arterial pressure of the patient and the intrinsic resistance of the circuit (determining the blood flow); the UF was determined by the hydrostatic pressure drop inside the filter and the negative suction provided by the UF column from the patient level to the ground. As a consequence, patients with low blood pressure and/or low cardiac output achieved the lowest clearances but were able to self-limit UF. When peristaltic pumps were added to the extracorporeal circuit, veno-venous hemofiltration became feasible. Subsequently, fluid delivery systems and UF control mechanisms were implemented allowing dialysate and replacement solutions to be delivered with acceptable accuracy. Higher clearances were finally possible because of the ability to provide increased flow rates. Although clearly improved, RRT machines were still inaccurate, and safety and performance were still a challenge. It soon became evident that an ideal extracorporeal circuit requires continuous pressure measurements at different levels (inlet and outlet of vascular access, inlet and outlet of the filter and UF ports) ^4^.

Currently, third/fourth-generation machines are designed to meet the dialysis dose requirements and the strict safety features that are recommended in every modern ICU ^5^. Contemporary devices, equipped with 4 to 5 roller pumps, 3 to 4 scales and pressure sensors, allow a fluid load from 20 to 40 kg in order to reduce nursing workload. In addition, maximal flow rates have increased up to about 450 mL⁄min for the blood pump, 8–10 L⁄hr for the dialysate⁄replacement pumps, and 20–25 L⁄hour for the effluent pump. Mechanical implementation has been associated with a huge electronic evolution: interfaces have been implemented with wide screens and clear alarm signals and warnings, and circuit pressure trends are now visualized. Furthermore, during continuous RRT (CRRT), now flexible and safe, modalities can be switched in order to tailor them to the patients’ need. Modern RRT filters, a key component of the system, are composed of groups of hollow fibers with a range of surface areas (from 0.1 to over 2 m ^2^) in order to meet the need of differently sized patients. Such fibers have a generally high porosity (30–50 A°) with a pore cutoff size of 30 kDa and are used for both diffusive and convective treatments. Polyacrylonitrile, polysulphone and poly(methyl methacrylate) (PMMA) are the most commonly used membranes and allow a high UF coefficient (over 20 ml/h/mmHg) and high diffusive and convective performance. Biocompatibility (the change in blood factors induced by membrane/blood contact) is considered the most important quality of these RRT membranes.

### Diffusion and convection

**Figure 1.**
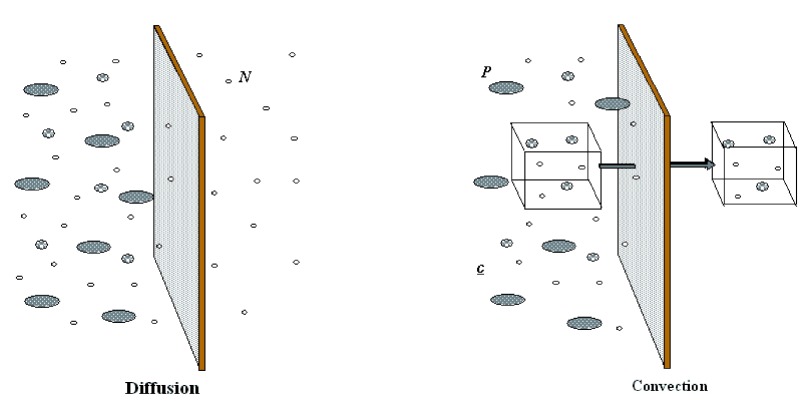
Diffusion and convection are schematically represented. During diffusion solutes flux (Jx) is a function of: solutes concentration gradient (dc) between the two sides of the semi-permeable membrane, temperature (T), diffusivity coefficient (D), membrane thickness (dx) and surface area (A) according to the following equation:     Jx = D T A (dc/dx) Convective flux of solutes (Jf) requires instead a pressure gradient between the two sides of the membrane (transmembrane pressure TMP), that moves a fluid (plasma water) with its « crystalloid » content (a process called ultrafiltration, whose entity is also dependent on membrane permeability coefficient (Kf). Colloids and cells will not cross the semipermeable membrane, depending on the pores’ size.     Jf = Kf × TMP

Renal replacement consists of blood purification by semi-permeable membranes. Blood flows into hollow fibers composed of porous biocompatible synthetic material. A wide range of substances (water, urea, low, middle and high molecular weight solutes) can be transported across such membranes, from the blood to the effluent side of the hollow fibers, by the mechanism of diffusion (solutes) and convection (water and solutes) ( Figure 1).

**Figure 2.**
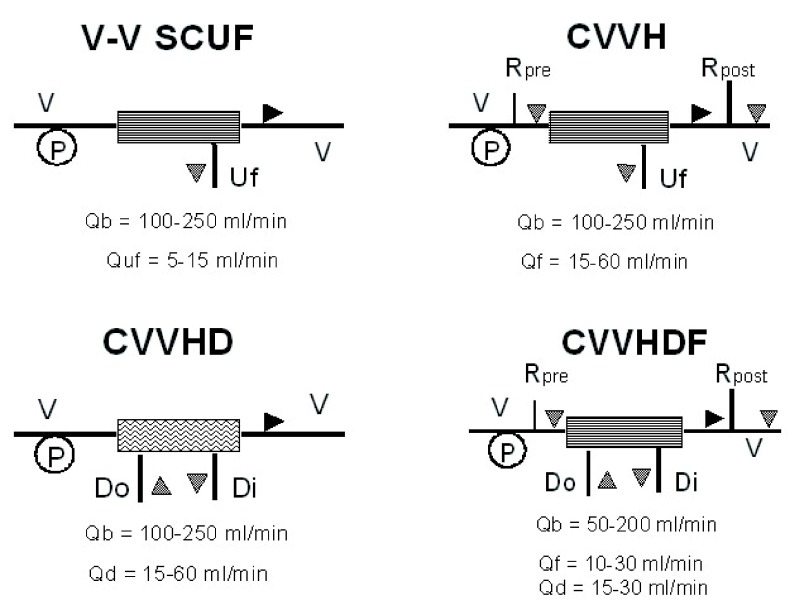
Schematic representation of most common continuous RRT set-ups. Black triangle represents blood flow direction; gray triangle indicates dialysate/replacement solutions flows. V-V: veno-venous; Uf: ultrafiltration; Rpre: replacement solution prefilter; Rpost: replacement solution postfilter; Do: dialysate out; Di: dialysate in; Qb: blood flow; Quf: ultrafiltration flow; Qf: replacement solution flow; Qd: dialysate solution flow.

Dialysis is based on the diffusion principle: a dialytic solution flows through the filter counter current to blood flow in order to maintain the highest solute gradient from inlet to outlet port. Diffusion is the solute transport method applied during intermittent hemodialysis (IHD) and continuous veno-venous hemodialysis (CVVHD) ( Figure 2). During diffusion, the movement of solutes depends on their tendency to reach the same concentration on each side of the membrane, allowing the passage of solutes from the compartment with the highest concentration to the compartment with the lowest concentration. Other components of the semi-permeable membrane that affect diffusion include thickness and surface area, dialysate temperature, and diffusion coefficient.

During convection, solutes are transported across a semi-permeable membrane by UF (water transfer across the membrane). In other words, as the solvent (plasma water) is pushed (ultrafiltered) across the membrane according to the TMP, solutes are carried with it, as long as the porosity of the membrane allows the molecules to be sieved from the blood. Convection is applied during continuous veno-venous hemofiltration (CVVH) while the combination of both convection and diffusion configures continuous veno-venous hemodiafiltration (CVVHDF) ( Figure 2).

The UF rate (Q _UF_) in CAVH systems was governed by the membrane UF coefficient (Km) and the TMP gradient generated by the pressures on both sides of the hollow fiber according to the following formula:

Q _UF_ = Km * TMP

In modern RRT machines Q _UF_ is regulated by a pump and, consequently, it is constantly maintained regardless of whether the filter is “fresh” (when UF occurs with low TMP) or clogging (in which case a progressive secondary increase of TMP is observed). In fact, as molecules cleared during convection are physically dragged to the UF side, the protein layering that progressively clogs the fiber pores significantly limits solute transport ^6^. A peculiar membrane capacity, defined as adsorption, has been shown to play a major role in higher molecular weight toxins ^7^; however, membrane adsorptive capacity is generally saturated within the first few treatment hours. This observation explains the minimal impact of the adsorption component on solute clearance ^8^. An exception on this rule is made by high-mobility group box 1 protein (HMGB-1), as this major sepsis key mediator can be significantly removed (more than 90%) by adsorption through an acrylonitrile-treated surface (AN69-ST) and PMMA membranes ^9^. However the clinical relevance of this molecule clearance remains to be ascertained. As UF proceeds and plasma water and solutes are filtered from blood, hydrostatic pressure within the filter declines and the effect of oncotic pressure increases because blood concentrates and hematocrit increases. The fraction of plasma water that is removed from blood during UF is called the filtration fraction and should be kept in the range of 20–25% in order to avoid equalization of the oncotic pressure to the TMP and filtration/pressure equilibrium. Finally, replacing the plasma water removed through the filter with a substitution solution completes the hemofiltration process and purified blood is returned to the patient. When the substitution fluid is administered after the filter it is referred to as post-dilution HF. When the substitution solution is infused before the filter it is referred to as pre-dilution HF. While post-dilution allows a urea clearance equivalent to therapy delivery (see below), pre-dilution, in spite of theoretical reduced solutes clearances, allows prolonged circuit lifespan by reducing hemoconcentration and protein caking effects within filter fibers. The difference between the volume of ultrafiltered plasma water and reinfused substitution solution gives the net UF, which is the fluid that is eventually removed from the patient for fluid control. Net UF prescription is based on patient needs and can range from more than 1 L/h (pulmonary edema in a patient with congestive heart failure and diuretic-resistant AKI) to zero (sepsis with catabolic state increased creatinine levels and conserved diuresis). A net UF rate must be added to diffusion-based CRRT modalities in order to achieve fluid balance control since diffusion does not allow for water exchanges.

Interestingly, apart from the demonstration of different clearances of middle molecular weight solutes (i.e beta-2 microglobulin) provided by CVVH when compared to similar CVVHD doses ^8^, no study so far has shown that the application of hemofiltration, with respect to hemodialysis, improves hard outcomes (such as mortality, length of mechanical ventilation, length of hospital stay) ^10, 11^.

### RRT dose

RRT dose is a measure of the quantity of blood purified by “waste products and toxins” and is generally expressed as clearance (K). Clearance is defined as the amount of blood purified by a single solute in the unit of time and it is expressed as volume over time, as it represents the flow of “cleaned” blood. As these still incompletely known substances “to be purified” are difficult to measure and quantify, the operative view of RRT dose is generally reduced to the measure of the elimination of a representative marker solute. Unfortunately, the marker solute does not represent all the solutes that accumulate during AKI because kinetics and volume of distribution are different for each solute and its removal during RRT is not necessarily representative of the removal of other solutes. However, since single solute marker assessment of dialysis dose appears to be related to patient outcome ^12^, urea and creatinine, due to their significant accumulation during AKI and the ease of their routine daily blood determination, are generally used as reference solutes for measuring renal replacement clearance during either chronic or acute dialysis.

During RRT, clearance depends upon blood flow rate (Qb), substitution flow rate (Qf) or dialysis flow (Qd), solute molecular weights, and hemodialyzer type and size. Qb is mainly dependent upon vascular access and the operational characteristics of utilized machines in the clinical setting. Qf is strictly linked to Qb, during convective techniques, by filtration fraction. Filtration fraction does not limit Qd, but when Qd/Qb ratio exceeds 0.3, dialysate will not be completely saturated with blood diffusing solutes. When UF is applied, molecules are dragged with plasma water through the filter pores according to their sieving coefficient (SC); the SC is calculated as the effluent/plasma concentration ratio of the target molecule. When the SC is 1, as in the case of small molecules (below 12 kD, such as creatinine and urea), the same solute concentration is found in the two sides of the hollow fiber. A SC value of 0 means that the molecule is not filtered (i.e. albumin, hemoglobin, etc). K during convection is measured by the product of Qf multiplied by the SC; hence, there is a linear relationship between K and Qf, the SC being the changing variable for different solutes. During diffusion, the linear relationship is lost when Qd exceeds about 1/3 of Qb.

One of the crucial merits of specific dose prescription, calculation, and delivery is the avoidance of underdialysis and the improved monitoring and awareness of effective delivered therapy.

### Continuous vs intermittent RRT

Blood purification can be achieved in the ICU both by continuous and intermittent RRT. In theory, during continuous RRT, the treatment is kept running 24 hours a day, seven days a week. During intermittent RRT renal support is delivered in intermittent sessions lasting (depending on center preferences, protocols, and the patient’s clinical status) 3–6 hours, typically three times per week (or depending on specific needs). Currently, about 80% of critically ill patients are treated with continuous RRT. However, due to the absence of significant differences in outcome deriving from the application of continuous vs intermittent RRT, no specific recommendation is provided by the major critical care societies, and the choice is mainly left to institutional protocols and expertise. Apart from evidence in large clinical trials, more gentle RRT application is generally better tolerated in hemodynamically unstable, critically ill patients with severe AKI. Furthermore, since the occurrence of intradialytic hypotension is proportional to the net Quf rate, it is possible to prescribe a lower net Quf rate when the treatment is applied over 24 hours as compared to a quick 3-hour session. Recently, the Surviving Sepsis Campaign guidelines for management of severe sepsis and septic shock ^13^ concluded that, based on present scientific evidence, continuous RRT should be considered equivalent to IHD for treatment of AKI. Vinsonneau and colleagues ^14^ conducted a large, prospective, randomized multicenter study in 21 ICUs over a 3.5-year period. The primary end point was the 60-day mortality following the randomization of 360 patients with AKI to either CVVHDF or IHD and no difference was found in 28, 60 and 90-day mortality between the two groups. Hence, according to the results, the study investigators concluded that IHD can be delivered safely to critically ill patients. Unfortunately, delivered dose in both arms was not controlled for in the trial design. The accompanying editorial ^15^ emphasized that the advantages of continuous therapies are particularly significant when therapy downtime is minimized, in order to enhance the low intensity, smooth and continuous effects of plasma K. However, Vinsonneau *et al*.’s findings have been confirmed repeatedly by other studies ^16, 17^. One of the reasons for the lack of hard outcome differences between intermittent and continuous techniques could be because IHD has become safer and more efficacious ^18^. Alternatively, a liberal application of CRRT (including extended and probably wrong indications) can cause adverse effects as discussed later below.

Hybrid techniques, which combine the advantages of both continuous and intermittent modalities, may represent an interesting compromise. Although a variety of names have been given to hybrid techniques (see the nomenclature section) ^19– 22^ depending on variations in schedule and type of solute removal (convective or diffusive), they all attempt to provide a gentle, prolonged and more feasible extended IHD, with all the advantages of discontinuous treatment (less need for anticoagulation, increased patient mobility, easier possibility of fitting prescribed schedules without downtime). These techniques generally have shown good results in terms of hemodynamic tolerance and adequacy of dialytic dosage ^23^. Baldwin and coworkers compared 3 consecutive days of CVVH with a similar period of extended daily dialysis with filtration ^23^. No significant difference was found between the two therapies as far as urea or creatinine levels and electrolyte and acid–base control. Interestingly, after 3 days of treatment, there was a mild but persistent metabolic acidosis in the extended dialysis group, but in the CVVH group hypophosphatemia was described. Advantages and disadvantages of IHD, CRRT and hybrid techniques, respectively, are depicted in Table 1.

**Table 1.** Intermittent vs Continuous vs Extended Dialysis.

	Advantages	Disadvantages	Contraindications
**Intermittent** **Hemodialysis (IHD)**	• Short duration • No/short/less anticoagulation (reduced risk of bleeding). • Higher efficiency for immediate small water-soluble removal (life-threatening hyperkalemia) • Less bed rest • Flexibility of use: machines can be used in an extended protracted mode (increase in efficacy) • Bags cost saving	• Technical skills (trained personnel) and technical infrastructure (dedicated areas with water connection) • Clearance rebound • Hemodynamic impact/instability • Potential higher risk of dialysis dependence	• Traumatic brain injury
**Continuous Renal** **Replacement Therapy** **(CRRT)**	• Hemodynamic stability (less cardiovascular impact) → higher potential recovery of kidney function • ICU staff may handle these treatments autonomously • Superior solute removal and volume control (in a 24 hours lasting session) • Administration of parenteral nutrition fluids	• Downtime may impair efficiency • Continuous systemic (heparin) or regional (citrate) anticoagulation (higher risk of patient’s bleeding or filter clotting) • Bed rest is necessary • Higher cost • Lower efficiency than IHD (hyperkalemia) • Risk of hypothermia	• Patients needing mobilization
**Sustained Low-Efficiency** **Daily Dialysis (SLEDD)** **or Prolonged intermittent** **RRT (PIRRT)**	• Easy • Good flexibility of sessions administration (6–12 hours or overnight treatment) • Higher possibility for patient mobility • Hemodynamic stability • Relatively low anticoagulation requirement • Bags cost saving	• Technical skills (trained personnel) and technical infrastructure (dedicated areas with water connection) • Hypophosphatemia • Hypothermia • Low efficiency	• None

In conclusion, intermittent and continuous therapies, when applied by expert centers, may appear similar where hard outcomes are concerned. As far as long-term RRT outcomes are concerned, however, recent reports indicate that RRT survivors treated by IHD might have a lower chance of recovering pre-morbid kidney function and have an increased risk of remaining dialysis-dependent at hospital discharge ^24^.

### Anticoagulation

The contact between blood and artificial surfaces induces activation of the coagulation cascade, resulting in filter and/or circuit clotting and the need for anticoagulation ^25– 29^. Anticoagulation strategy depends on the type of RRT and is often needed for continuous therapies due to the increased exposure to the blood-artificial surface. Aims of anticoagulation are: maintenance of extracorporeal circuit and dialyzer patency; reduction of downtime that might have a clinical impact in the overall RRT clearance; reduction of treatment cost by the utilization of less material; and achievement of the above aims while minimizing risks for the patient. Several technical features of the RRT circuit are likely to affect the success of any anticoagulant approach: vascular access has to be of adequate size; tube kinking should be avoided; blood flow rate should exceed 100 ml/min; pump flow fluctuations must be prevented; and the venous bubble trap, where air/blood contact occurs, must be accurately monitored. Furthermore, plasma filtration fraction should be kept as far as possible below 20% and, when possible, pre-dilution hemofiltration should be selected. There is evidence that, when circuit set-up is perfectly optimized, anticoagulants are only a relatively minor component of circuit patency. When patients have altered coagulation, thrombocytopenia, or active bleeding (e.g. after trauma or surgery), RRT can be safely performed without anticoagulation ^25^. Lastly, regional citrate anticoagulation (RCA) can be safely used nowadays not only in patients with bleeding risks but also in patients without bleeding risks, according to the current KDIGO guidelines ^30^. Extensive training is needed regarding the metabolic side effects of citrate before embarking upon routine citrate anticoagulation. In recent years, new commercially available citrate solutions together with adapted CRRT machines have rendered the technique safer and easier to use ^31^.

Different methods for anticoagulation are summarized in Table 2.

**Table 2.** Anticoagulation strategies.

Drug	Indication	Contra	Comment
No anticoagulation	High risk bleeding profile	Relative shorter circuit lifespan	RRT can be safely performed without anticoagulant
UFH	Routine	HIT	Antidote is available (protamine). Monitoring: aPTT. Serum antithrombin levels have to be optimized
LMWH	Routine (alternative to UH)	HIT	Better bioavailability than UFH
PGI2	Very short circuit lifespan	Hypotension	Potent inhibitor of platelet aggregation with a short half- life. Hypotension might occur. Its high cost and harmful side effects might limit the use
Citrate	Routine/Very short circuit lifespan	Hypocalcemia	Regional anticoagulation. Calcium is chelated in the filter and then calcium chloride is infused back to the patient to maintain normocalcemia. Excellent filter patency. Relative drawbacks include the risk for hypocalcemia, metabolic alkalosis/acidosis, and the cumbersome replacement/dialysate fluid preparation
Danaparoid	HIT		Insufficient data available
Argatroban	HIT		Insufficient data available
Irudine	HIT		Insufficient data available
Nafamostat mesilate	HIT		Insufficient data available
Heparin coated circuits	Routine		Insufficient data available

**Abbreviations**: RRT, renal replacement therapy; UHF, unfractioned heparin; HIT, heparin-induced thrombocytopenia; aPTT, activated prothrombin time; LMWH, low molecular weight heparin; PGI2, prostacyclin.

## Clinical applications

### Indications to start RRT

Regardless of RRT technique used, the following clinical variables are typically compromised in the critically ill patient with AKI: fluid status and tissue edema, hemodynamics, acid–base and electrolyte equilibrium, protein-rich nutritional support, phosphate and calcium balance, and infection control.

Currently, a broader concept of “timely intervention” is generally accepted. When oliguria results in impairment of one or more of the above clinical variables, RRT should be instituted rapidly in order to avoid fluid overload and congestion. The only urgent indications to perform dialysis are pulmonary edema refractory to high dose diuretics, rapidly increasing hyperkalemia, severe refractory acidosis, symptoms/signs of uremia, and specific drug intoxications. Critically ill patients, especially if they are oliguric or anuric, typically gain weight from water accumulation and large volumes of intravenous fluids. In such patients, water removal is indicated for the achievement of a negative daily fluid balance, which has been associated with multiorgan function improvement (i.e. at the pulmonary, cardiac and renal level) in observational and retrospective studies ^32, 33^. Furthermore, a slow continuous RRT with fluid removal over 24 hours is better able to manage the critically ill patient’s needs; in case of increased nutritional administration, fluids deriving from parenteral drugs or hemoderivates transfusion, Q _UF_ can be easily tailored on an hourly base ^34^. Conversely, rapid (or, worse, intermittent) ultrafiltration of body water may lead to acute hypovolemia and subsequent hypotension, since refilling from the interstitial compartment is slow and steady due to hydrostatic and osmotic pressures ^35^. Since no effective clinical monitoring is currently available to “measure” fluid overload and the amount of fluid excess to be removed, clinical expertise in critical care nephrology (and possibly a multidisciplinary approach) is essential for adequate management of fluid removal ^36^.

As for the exact timing for starting RRT, a definition of timing is currently not available. Timing can be considered as a synonym for “indication” and then one can start CRRT early or late depending on how severe (or conventional) the indication is (e.g. creatinine level or potassium level or the presence of sepsis) ^37^. Otherwise, timing can be considered as the time elapsed between any established indication to start and the effective inception of the dialytic session. A recent retrospective study ^38^ confirmed that crude 90-day mortality of patients with RRT started after “classic indications” (identified as hyperkalemia, severe acidosis, urea above 100 mg/dl, oliguria or anuria and fluid overload with pulmonary edema) was significantly higher than in patients with “pre-emptive” RRT (initiated without any conventional indication): adjusted odds ratio, 2.05; 95% CI, 1.03 to 4.09. Interestingly, also patients with classic RRT but a delayed start (>12 hours from indication) showed higher crude mortality compared with patients with classic RRT that started early due to urgent indications (<12 hours from indication); this association persisted after adjustment for known confounders (odds ratio, 3.85; 95% CI, 1.48 to 10.22). Due to the retrospective nature of this study, it is not clear how effectively comparable the two populations (classic vs pre-emptive or early vs delayed) are. The Canadian Critical Care Trials Group recently concluded the first pilot trial aiming to prospectively evaluate the feasibility of a protocol-driven accelerated RRT initiation ^39^. This interesting complex trial showed how difficult it would be to conduct a large multicenter prospective trial attempting to randomize two AKI populations only differing by the time elapsed from RRT indication to treatment start. In fact, in the pilot trial, a large number of patients had to be excluded after provisional eligibility per protocol design: those deemed by the intensivist and nephrologist in charge as requiring urgent RRT or deferral of RRT indication. This excluded cohort might unfortunately represent a sample of patients whose outcomes are potentially affected by pre-emptive or delayed RRT start. However, in the analysis of the enrolled 101 patients, the authors succeeded in their primary outcome. In the accelerated arm, median time to RRT start was 7.4h. In the standard arm, 33 patients started RRT at a median of 31.6h from eligibility, and, interestingly, the other 19 did not receive any RRT (6 died and 13 recovered kidney function). Even though these preliminary results should be interpreted with caution, hard outcomes were not affected by acceleration of RRT start (mortality was 38% in the accelerated and 37% in the standard arm).

### Prescription and maintenance of RRT

From a clinical standpoint, the effects of RRT dose have been systematically evaluated in the last 10 years. After the milestone trial from the group in Vicenza back in 2000 ^40^, CVVH dose has been indexed for the first time to patients’ body weight (mL/Kg/h), in order to highlight that this variable is of high importance in AKI patients. Two large, multicenter, randomized controlled studies published in 2009 (the randomized evaluation of normal versus augmented level (RENAL) replacement therapy study ^41^ and in 2008, the VA/NIH Acute Renal Failure Trial Network (ATN) study ^42^) finally clarified the concept of optimal dialysis dose. These fundamental trials were conceived to test the hypothesis concerning the impact of “intensive” RRT on hard outcomes (namely mortality and ICU stay) when compared to “less intensive” renal support. The RENAL study was conducted exclusively with continuous therapies (as this is the standard in Australia) and compared 25 mL/Kg/h CVVHDF to 40 mL/Kg/h. Using a different approach, the ATN study, conducted in North America, considered 20 mL/Kg/h CVVHDF or thrice weekly intermittent dialysis as the control group and compared it to 35 mL/Kg/h CVVHDF or daily IHD as the intensive arm. Apart from methodological differences, both studies confirmed that “intensive” RRT does not improve patient outcomes, and survival (although different between Australia’s and United States’ centers) was similar among compared arms. Based on the results of those trials, the accepted dose of RRT is considered to be within the range of 25–35 mL/Kg/h for CRRT and/or thrice weekly IHD with a Kt/V (see table on nomenclature) of 1.3.

Clearly, clinical effects of RRT dose are not limited to urea and excess body water control. Oligo anuric patients often suffer from mild acidemia secondary to increased unmeasured anions (strong ion gap – SIG - 12.3 mEq/l), hyperphosphatemia, and hyperlactatemia. This acidosis is attenuated by the alkalizing effect of hypoalbuminemia. The effect on acid–base balance of IHD and CVVHDF has been evaluated ^43^: metabolic acidosis is common in both groups and both techniques correct metabolic acidosis; however, the rate and degree of correction may significantly differ between continuous and intermittent techniques. In the same study ^43^ CVVHDF was shown to normalize metabolic acidosis more rapidly and more effectively during the first 24 hours than IHD. IHD was also associated with a higher incidence of metabolic acidosis as compared to CVVHDF during the subsequent 2-week treatment period. Accordingly, continuous RRT could be considered physiologically superior to IHD in the correction of metabolic acidosis. In a comparison between CVVH and peritoneal dialysis, all patients receiving CVVH achieved correction of acidosis by 50 hours of treatment, whereas only 15% of those randomized to peritoneal dialysis achieved such correction (P < 0.001) ^44^. Despite the results of these studies, correction of acidosis by RRT has not revealed any specific impact on outcomes.

Although safety features of CRRT machines have evolved, the possibility that CRRT may confer increased risk should not be overlooked ^45^. In fact, as with any type of continuous extra corporeal therapy, CRRT often requires continuous anticoagulation therapy, which can increase the bleeding risk in case of heparin use or metabolic derangements in case of citrate use. Conversely, clotting of the extracorporeal circuit also occurs frequently with CRRT, which might contribute to blood loss and could exacerbate anemia in critically ill patients. The increased solute transfer associated with the use of CRRT might enhance removal of amino acids, vitamins, catecholamines, and other solutes. As alluded to before, therapy downtime (the period when a prescribed CRRT has not run due to unplanned interruptions) should be carefully controlled, possibly limited and eventually compensated, because it might significantly impact dialysis delivery ^46– 47^. Following this path, it might be speculated that the quality of care and the specific dialysis monitoring is likely to be superior when a dialysis nurse is attending the treatment session ^48^. In order to meet the safety requirement, the new generation of CRRT machines has been implemented with a strict safety profile limiting dangerous side effects of dialytic treatments. In any case, ICU staff training is mandatory before starting the routine utilization of such monitors.

A synopsis of RRT prescription is also presented in Table 3.

**Table 3.** Algorithm for RRT prescription.

Clinical variables	Operational variables	Setting
Fluid balance	Net Ultrafiltration	A continuous management of negative balance (100–300 ml/h) is preferred in hemodynamically unstable patients.
Adequacy and Dose	Clearance/Modality	25–35 ml/Kg/h for CRRT, consider first CVVHDF (even if no evidence is available about which modality is better). If IHD is selected, at least a Kt/V of 1.3 on alternate days should be targeted even if fluid balance can be adequately managed only by everyday dialysis.
Acid–Base	Solution Buffer	Bicarbonate buffered solutions are preferable to lactate buffered solutions in case of lactic acidosis and/or hepatic failure.
Electrolyte	Dialysate/Replacement	Consider solutions without K ^+^ in case of severe hyperkalemia. Manage accurately MgPO _4_.
Timing	Schedule	Early and “adequate” RRT is suggested even if no specific recommendation is available.
Protocol	Staff/Machine	Well-trained staff should routinely utilize RRT monitors according to predefined institutional protocols.

**Abbreviations**: CVC, central venous catheter; S-G, Swan Ganz catheter; EKG, electrocardiogram; CRRT, continuous renal replacement therapy; CVVHDF, continuous veno-venous hemodiafiltration; IHD, intermittent hemodialysis; MgPO _4_, magnesium phosphate.

### Indications to stop RRT

In the specific setting of “weaning from RRT”, no good evidence exists at present and it is unlikely to be the case in the near future. Nevertheless, some insights may be gleaned from recent available literature. An interesting report from the Beginning and Ending Supportive Therapy for the Kidney (BEST Kidney) investigators described current practice for the discontinuation of CRRT in order to identify variables associated with successful discontinuation and whether the approach to discontinue CRRT therapy affected patient outcomes ^49^. Statistical analysis identified urine output and creatinine as significant predictors of successful cessation. The predictive value of urine output was negatively affected by the use of diuretics. Risk factors for re-dialysis were also analyzed ^50^: the 94 postoperative patients analyzed by these authors were considered free from RRT if after at least 30 days they did not require dialysis. Successful weaning from RRT was correlated with Sequential Organ Failure Assessment (SOFA) score, age, dialysis duration and, again, urine output. Interestingly, out of the patients who remained “RRT-free” for 5 days after RRT discontinuation, more than two-thirds (20) remained RRT-free for up to 30 days.

As a general recommendation, before weaning from RRT, physicians should wait for adequate urine output (without diuretic therapy) and optimized creatinine values. Once renal function appears close to the baseline or “pre-AKI” level, it seems reasonable to interrupt the treatment without any specific weaning protocol. Future trials, including the identification of new biomarkers, are needed to design novel weaning protocols.

## Conclusions

In recent years, great technological improvements have been made in the manufacturing of extracorporeal circuits, rendering them easier to use, safer and more efficient for long-term support. Modern RRT systems can be managed in the ICU by one bedside nurse who is trained and experienced in circuit management, and it is now possible to treat patients for several weeks, or even months, without major complications. Thanks to technology development, the possibility of removing “waste products” is currently open to several molecules, including middle-sized ones, different from creatinine. It is now becoming a reality to integrate multiple devices into a single user-friendly machine for CO _2_ clearance, hemoperfusion, plasma-filtration and adsorption responding to different medical needs ^51– 58^. Finally, advances in information technology should allow the fully integrated extracorporeal blood purification system to be connected to all electronic therapeutic devices, from simple syringe pumps to CRRT machines, in order to ultimately lead to an ‘‘artificial organ’’ in a more complete sense ^59^. In such a detailed and diversified technological world it is of utmost importance that communication among practitioners (physicians, nurses, technicians, researchers) is homogeneous and widely accepted. In light of this, nomenclature is a crucial aspect concerning RRT (please see Table 4 for a list of terminology and its significance). It is extremely important to avoid a sort of “Tower of Babel” effect by sharing a common language ^60^.

**Table 4.** Nomenclature.

Nomenclature	Description
Intermittent hemodialysis (IHD)	A prevalently diffusive treatment in which blood and dialysate are circulated in counter current mode and, generally, a low permeability, cellulose-based membrane is employed. Dialysate must be pyrogen free but not necessarily sterile, since dialysate-blood contact does not occur. The UF rate is equal to the scheduled weight loss. This treatment can be typically performed 4 (to 6) hours thrice weekly or daily. Qb: 150–450 ml/min Qd: 300–600 ml/min.
Kt/V	This is an adimensional number utilized to express clearance during IHD. The numerator expresses intensity or clearance (K) per time (Kt) and denominator indicates the solute volume of distribution (V): in theory, a Kt/V of 1 implies that a dialytic session delivered with a certain K of a specific solute (generally urea) for a determined period of time (t) has completely removed the marker solute from patient volume of distribution (V). In practice, generation rate of the marker solute (and other complex factors) avoids blood concentration of the given solute to be zeroed.
Peritoneal dialysis (PD):	A predominantly diffusive treatment where blood, circulating along the capillaries of the peritoneal membrane, is exposed to dialysate. Access is obtained by the insertion of a peritoneal catheter, which allows the abdominal instillation of dialysate. Solute and water movement is achieved by the means of variable concentration and tonicity gradients generated by the dialysate. This treatment can be performed continuously or intermittently.
Slow continuous ultrafiltration (SCUF):	Technique where blood is driven through a highly permeable filter via an extracorporeal circuit in veno-venous mode. The ultrafiltrate produced during membrane transit is not replaced and it corresponds to weight loss. It is used only for fluid control in overloaded patients (i.e. congestive heart failure resistant to diuretic therapy). Qb: 100–250 ml/min. Quf: 5–15 ml/min ( Figure 2).
Continuous veno-venous hemofiltration (CVVH):	Technique where blood is driven through a highly permeable filter via an extracorporeal circuit in veno-venous mode. The ultrafiltrate produced during membrane transit is replaced in part or completely to achieve blood purification and volume control. If replacement fluid is delivered after the filter, the technique is defined as post-dilution hemofiltration. If it is delivered before the filter, the technique is defined as pre-dilution hemofiltration. The substitution fluid can also be delivered both pre and post filter. Clearance for all solutes is convective and equals UF rate. Qb: 100–250 ml/min. Quf: 15–60 ml/min ( Figure 2).
Continuous veno-venous hemodialysis (CVVHD):	Technique where blood is driven through a low permeability dialyzer via an extracorporeal circuit in veno-venous mode and a counter current flow of dialysate is delivered on the dialysate compartment. The ultrafiltrate produced during membrane transit corresponds to patient’s weight loss. Solute clearance is mainly diffusive and efficiency is limited to small solutes only. Qb: 100–250 ml/min. Qd: 15–60 ml/min ( Figure 2).
Continuous veno-venous hemodiafiltration (CVVHDF):	Technique where blood is driven through a highly permeable dialyzer via an extracorporeal circuit in veno-venous mode and a countercurrent flow of dialysate is delivered on the dialysate compartment. The ultrafiltrate produced during membrane transit is in excess of the patient’s desired weight loss. A replacement solution is needed to maintain fluid balance. Solute clearance is both convective and diffusive. Qb: 100–250 ml/min. Qd: 15–60 ml/min. Qf: 15–60 ml/min ( Figure 2).
Hybrid Techniques	Sustained low-efficiency extended daily dialysis (SLEDD), prolonged daily intermittent RRT (PDIRRT), extended daily dialysis (EDD), extended daily dialysis with filtration (EDDf), extended IHD.
Hemoperfusion (HP):	Blood is circulated on a bed of coated charcoal powder to remove solutes by adsorption. The technique is specifically indicated in cases of poisoning or intoxication with agents that can be effectively removed by charcoal. Polymixin hemoperfusion has been attempted for endotoxin removal in gram-negative septic AKI patients ^51^. This treatment may cause platelet and protein depletion.
Plasmapheresis (PP):	A treatment that uses specific plasmafilters. Molecular weight cut-off of the membrane is much higher than that of hemofilters (100–1000 kDa): plasma as a whole is filtered and blood is reconstituted by the infusion of plasma products such as frozen plasma or albumin. This technique is performed to remove proteins or protein-bound solutes.
High flux dialysis (HFD):	A treatment that utilizes highly permeable membranes in conjunction with an UF control system. Due to the characteristics of the membrane, UF occurs in the proximal part of the filter that is counterbalanced by a positive pressure applied to the dialysate compartment: this causes a phenomenon called backfiltration in the distal part of the filter. Hence, diffusion and convection are combined, but, thanks to the use of a pyrogen-free dialysate, replacement is avoided.
High volume hemofiltration (HVHF):	HVHF is defined as continuous high-volume treatment with an effluent rate of 50 to 70 ml/kg/hour (for 24 hours per day) or intermittent very high-volume treatment with an effluent rate of 100 to 120 ml/kg/hour for a 4- to 8-hour period followed by conventional renal-dose hemofiltration ^61^. Clinical benefits of HVHF have been recently questioned.
High cut-off hemofiltration or Hemodialysis	A technique aimed at removing inflammatory mediators (e.g. cytokines) in septic patients. HCO membranes are porous enough to achieve the removal of larger molecules (approximately 15 to 60 kD) by diffusion. Its ability to remove cytokines in *ex vivo* and *in vivo* studies has now been shown to be greater than that of any other technology so far ^52^ and has increased survival in experimental models of sepsis ^53^. HCO therapy seems to have beneficial effects on immune cell function and preliminary human studies using intermittent hemodialysis with HCO membranes have confirmed its ability to remove marker cytokines IL-6 and IL-1 receptor antagonist, with a decreased dosage of norepinephrine in patients with sepsis ^54^. Predictably, albumin losses are significant, but may be attenuated by using HCO membranes in a diffusive rather than convective manner while still preserving the effect on cytokine clearance.
Plasma Therapy	The term “plasma therapy” actually encompasses two therapies: plasma-adsorption and plasma exchange. In plasma-adsorption, plasma separated from blood cells flows along one or more columns that contain different adsorbents, after which the processed plasma is re-infused back to the patient. Plasma exchange is a single-step process in which blood is separated into plasma and cells and the cells are returned back to the patient while the plasma is replaced with either donor plasma or albumin. With respect to sepsis, it has been argued that plasma therapy is most likely to be effective in patients with sepsis-associated thrombotic microangiopathy ^55^.
Coupled plasma filtration adsorption (CPFA)	CPFA uses a resin cartridge inserted downstream from a plasma filter, improving the removal of nonspecific septic mediators with promising results in early small trials ^56, 57^ although these have been recently doubted ^62^. CPFA is aimed at non-selectively reducing the circulating levels and activities of both pro- and anti-inflammatory mediators during sepsis and multiorgan failure. In order to overcome the shortcomings of plasma filtration and improving the removal efficiency, CPFA uses a specific sorbent cartridge inserted in series with, but downstream to, the plasma filter.
Blood purification therapies	Literature on therapeutic effects of blood purification therapies in septic patients is not univocal. However, a number of confounding factors make these studies not comparable. Careful patient stratification on microbiological and clinical characteristics of sepsis together with the identification of the optimal timing for specific interventions should be the starting points for clinical application to this complex category of patients. Further data from new studies are needed to better define the role of these advanced therapies in septic AKI-ICU patient.

**Abbreviations**: Qb, blood flow; Qd, dialysis flow; Qf, ultrafiltration rate; UF, ultrafiltration; kD, kiloDaltons; HCO, high cut-off; IL, interleukin; AKI, acute kidney injury; ICU, intensive care unit.

## References

[1] Martin GS: Sepsis, severe sepsis and septic shock: changes in incidence, pathogens and outcomes. *Expert Rev Anti Infect Ther.* 2012;10(6):701–6. 10.1586/eri.12.50 22734959 PMC3488423

[2] Ricci Z Romagnoli S Ronco C: Renal support. *Minerva Anestesiol.* 2011;77(12):1204–15. 21623338

[3] Kramer P Wigger W Rieger J: [Arteriovenous hemofiltration: a new and simple method for treatment of over-hydrated patients resistant to diuretics]. *Klin Wochenschr.* 1977;55(22):1121–2. 10.1007/BF01477940 592681

[4] Ronco C Polaschegg HD: History and development of continuous renal replacement therapy. In: *Critical Care Nephrology: Expert Consult*– Edited by: Claudio Ronco MD, Rinaldo Bellomo MBBS(Hons) MD FRACP FCCP, John Kellum MD (Author). Philadelphia: Saunders Elseveier,2009;1323–1325.

[5] Ricci Z Bonello M Salvatori G: Continuous renal replacement technology: from adaptive technology and early dedicated machines towards flexible multipurpose machine platforms. *Blood Purif.* 2004;22(3):269–276. 10.1159/000078431 15148455

[6] Ricci Z Bellomo R Ronco C: Renal Replacement Techniques: Descriptions, Mechanisms, Choices and Controverises. In: *Critical Care Nephrology: Expert Consult*– Edited by: Claudio Ronco MD, Rinaldo Bellomo MBBS(Hons) MD FRACP FCCP, John Kellum MD (Author). Philadelphia: Saunders Elseveier,2009;1136–41. Reference Source

[7] Cole L Bellomo R Davenport P: Cytokine removal during continuous renal replacement therapy: an *ex vivo* comparison of convection and diffusion. *Int J of Artif Organs.* 2004;27(5):388–397. 15202816 10.1177/039139880402700507

[8] Ricci Z Ronco C Bachetoni A: Solute removal during continuous renal replacement therapy in critically ill patients: convection versus diffusion. *Crit Care.* 2006;10(2):R67. 10.1186/cc4903 16646985 PMC1550874

[9] Yumoto M Nishida O Moriyama K: *In vitro* evaluation of high mobility group box 1 protein removal with various membranes for continuous hemofiltration. *Ther Apher Dial.* 2011;15(4):385–93. 10.1111/j.1744-9987.2011.00971.x 21884474

[10] Friedrich JO Wald R Bagshaw SM: Hemofiltration compared to hemodialysis for acute kidney injury: systematic review and meta-analysis. *Crit Care.* 2012;16(4):R146. 10.1186/cc11458 22867021 PMC3580734

[11] Wald R Friedrich JO Bagshaw SM: Optimal Mode of clearance in critically ill patients with Acute Kidney Injury (OMAKI)--a pilot randomized controlled trial of hemofiltration versus hemodialysis: a Canadian Critical Care Trials Group project. *Crit Care.* 2012;16(5):R205. 10.1186/cc11835 23095370 PMC3682309

[12] Ricci Z Bellomo R Ronco C: Dose of dialysis in acute renal failure. *Clin J Am Soc Nephrol.* 2006;1(3):380–8. 10.2215/CJN.00520705 17699235

[13] Dellinger RP Levy MM Carlet JM: Surviving Sepsis Campaign: international guidelines for management of severe sepsis and septic shock: 2008. *Crit Care Med.* 2008;36(1):296–327. 18158437 10.1097/01.CCM.0000298158.12101.41

[14] Vinsonneau C Camus C Combes A: Continuous venovenous haemodiafiltration versus intermittent haemodialysis for acute renal failure in patients with multiple-organ dysfunction syndrome: a multicentre randomised trial. *Lancet.* 2006;368(9533):379–85. 10.1016/S0140-6736(06)69111-3 16876666

[15] Kellum J Palevsky PM: Renal support in acute kidney injury. *Lancet.* 2006;368(9533):344–5. 10.1016/S0140-6736(06)69084-3 16876645

[16] Guérin C Girard R Selli JM: Intermittent versus continuous renal replacement therapy for acute renal failure in intensive care units: results from a multicenter prospective epidemiological survey. *Intensive Care Med.* 2002;28(10):1411–8. 10.1007/s00134-002-1433-0 12373465

[17] Uehlinger DE Jakob SM Ferrari P: Comparison of continuous and intermittent renal replacement therapy for acute renal failure. *Nephrol Dial Transplant.* 2005;20(8):1630–7. 10.1093/ndt/gfh880 15886217

[18] Schortgen F Soubrier N Delclaux C: Hemodynamic tolerance of intermittent hemodialysis in critically ill patients: usefulness of practice guidelines. *Am J Respir Crit Care Med.* 2000;162(1):197–202. 10.1164/ajrccm.162.1.9907098 10903241

[19] Marshall MR Golper TA Shaver MJ: Urea kinetics during sustained low-efficiency dialysis in critically ill patients requiring renal replacement therapy. *Am J Kidney Dis.* 2002;39(3):556–570. 10.1053/ajkd.2002.31406 11877575

[20] Naka T Baldwin I Bellomo R: Prolonged daily intermittent renal replacement therapy in ICU patients by ICU nurses and ICU physicians. *Int J of Artif Organs.* 2004;27(5):380–387. 15202815 10.1177/039139880402700506

[21] Kumar VA Craig M Depner TA: Extended daily dialysis: A new approach to renal replacement for acute renal failure in the intensive care unit. *Am J Kidney Dis.* 2000;36(2):294–300. 10.1053/ajkd.2000.8973 10922307

[22] Kielstein JT Kretschmer U Ernst T: Efficacy and cardiovascular tolerability of extended dialysis in critically ill patients: a randomized controlled study. *Am J Kidney Dis.* 2004;43(2):342–349. 10.1053/j.ajkd.2003.10.021 14750100

[23] Baldwin I Naka T Koch B: A pilot randomised controlled comparison of continuous veno-venous haemofiltration and extended daily dialysis with filtration: effect on small solutes and acid–base balance. *Intensive Care Med.* 2007;33(5):830–5. 10.1007/s00134-007-0596-0 17384931

[24] Ricci Z Romagnoli S: Renal replacement therapy for critically ill patients: an intermittent continuity. *Crit Care.* 2014;18(2):115. 10.1186/cc13756 24670363 PMC3997812

[25] Tan HK Baldwin I Bedllomo R: Continuous veno-venous hemofiltration without anticoagulation in high-risk patients. *Intensive Care Med.* 2000;26(11):1652–1657. 10.1007/s001340000691 11193272

[26] Fiaccadori E Maggiore U Rotelli C: Continuous haemofiltration in acute renal failure with prostacyclin as the sole anti-haemostatic agent. *Intensive Care Med.* 2002;28(5):586–593. 10.1007/s00134-002-1249-y 12029407

[27] Kutsogiannis DJ Gibney N Stollery D: Regional citrate versus systemic heparin anticoagulation for continuous renal replacement in critically ill patients. *Kidney Int.* 2005;67(6):2361–2367. 10.1111/j.1523-1755.2005.00342.x 15882280

[28] Kim IB Fealy N Baldwin I: Insertion side, body position and circuit life during continuous renal replacement therapy with femoral vein access. *Blood Purif.* 2011;31(1–3):42–6. 10.1159/000322254 21160179

[29] Monchi M Berghmans D Ledoux D: Citrate vs. heparin for anticoagulation in continuous venovenous hemofiltration: a prospective randomized study. *Intensive Care Med.* 2004;30(2):260–265. 10.1007/s00134-003-2047-x 14600809

[30] KDIGO AKI Work Group: KDIGO clinical practice guideline for acute kidney injury. *Kidney Int (Suppl).* 2012;17:1–138.

[31] Jacobs R Honoré PM Bagshaw SM: Citrate Formulation Determines Filter Lifespan during Continuous Veno-Venous Hemofiltration: A Prospective Cohort Study. *Blood Purif.* 2015;40(3):194–202. 10.1159/000438820 26302765

[32] Payen D de Pont AC Sakr Y: A positive fluid balance is associated with a worse outcome in patients with acute renal failure. *Crit Care.* 2008;12(3):R74. 10.1186/cc6916 18533029 PMC2481469

[33] RENAL Replacement Therapy Study Investigators, Bellomo R Cass A: An observational study fluid balance and patient outcomes in the Randomized Evaluation of Normal vs. Augmented Level of Replacement Therapy trial. *Crit Care Med.* 2012;40(6):1753–60. 22610181 10.1097/CCM.0b013e318246b9c6

[34] Ronco C Ricci Z: Renal replacement therapies: physiological review. *Intensive Care Med.* 2008;34(12):2139–46. 10.1007/s00134-008-1258-6 18791697

[35] Gibney N Cerda J Davenport A: Volume management by renal replacement therapy in acute kidney injury. *Int J Artif Organs.* 2008;31(2):145–155. 18311730 10.1177/039139880803100207

[36] Ponce D Zorzenon Cde P dos Santos NY: Early nephrology consultation can have an impact on outcome of acute kidney injury patients. *Nephrol Dial Transplant.* 2011;26(10):3202–6. 10.1093/ndt/gfr359 21765052

[37] Ricci Z Ronco C: Timing, dose and mode of dialysis in acute kidney injury. *Curr Opin Crit Care.* 2011;17(6):556–61. 10.1097/MCC.0b013e32834cd360 22027405

[38] Vaara ST Reinikainen M Wald R: Timing of RRT based on the presence of conventional indications. *Clin J Am Soc Nephrol.* 2014;9(9):1577–85. 10.2215/CJN.12691213 25107952 PMC4152821

[39] Wald R Adhikari NK Smith OM: Comparison of standard and accelerated initiation of renal replacement therapy in acute kidney injury. *Kidney Int.* 2015;88(4):897–904. 10.1038/ki.2015.184 26154928

[40] Ronco C Bellomo R Homel P: Effects of different doses in continuous veno-venous haemofiltration on outcomes of acute renal failure: a prospective randomised trial. *Lancet.* 2000;356(9223):26–30. 10.1016/S0140-6736(00)02430-2 10892761

[41] RENAL Replacement Therapy Study Investigators, Bellomo R Cass A: Intensity of continuous renal-replacement therapy in critically ill patients. *N Engl J Med.* 2009;361(17):1627–38. 10.1056/NEJMoa0902413 19846848

[42] VA/NIH Acute Renal Failure Trial Network, Palevsky PM Zhang JH: Intensity of renal support in critically ill patients with acute kidney injury. *N Engl J Med.* 2008;359(1):7–20. 10.1056/NEJMoa0802639 18492867 PMC2574780

[43] Uchino S Bellomo R Ronco C: Intermittent versus continuous renal replacement therapy in the ICU: impact on electrolyte and acid–base balance. *Intensive Care Med.* 2001;27(6):1037–43. 10.1007/s001340100953 11497136

[44] Phu NH Hien TT Mai NT: Hemofiltration and peritoneal dialysis in infection-associated acute renal failure in Vietnam. *N Engl J Med.* 2002;347(12):895–902. 10.1056/NEJMoa020074 12239258

[45] Elseviers MM Lins RL Van der Niepen P: Renal replacement therapy is an independent risk factor for mortality in critically ill patients with acute kidney injury. *Crit Care.* 2010;14(6):R221. 10.1186/cc9355 21122146 PMC3219996

[46] Oudemans-van Straaten HM: Primum non nocere, safety of continuous renal replacement therapy. *Curr Opin Crit Care.* 2007;13(6):635–7. 10.1097/MCC.0b013e3282f161b2 17975382

[47] Kleger GR Fässler E: Can circuit lifetime be a quality indicator in continuous renal replacement therapy in the critically ill? *Int J Artif Organs.* 2010;33(3):139–46. 20383858 10.1177/039139881003300302

[48] Ricci Z Benelli S Barbarigo F: Nursing procedures during continuous renal replacement therapies: a national survey. *Heart Lung Vessel.* 2015;7(3):224–30. 26495268 PMC4593015

[49] Uchino S Bellomo R Morimatsu H: Discontinuation of continuous renal replacement therapy: a post hoc analysis of a prospective multicenter observational study. *Crit Care Med.* 2009;37(9):2576–2582. 10.1097/CCM.0b013e3181a38241 19623048

[50] Wu VC Ko WJ Chang HW: Risk factors of early redialysis after weaning from postoperative acute renal replacement therapy. *Intensive Care Med.* 2008;34(1):101–108. 10.1007/s00134-007-0813-x 17701162

[51] Early Use of Polymyxin B Hemoperfusion in the Abdominal Sepsis 2 Collaborative Group: Polymyxin B hemoperfusion in clinical practice: the picture from an unbound collaborative registry. *Blood Purif.* 2014;37(Suppl 1):22–5. 10.1159/000356835 24457492

[52] Uchino S Bellomo R Goldsmith D: Super high flux hemofiltration: a new technique for cytokine removal. *Intensive Care Med.* 2002;28(5):651–655. 10.1007/s00134-002-1261-2 12029417

[53] Lee PA Weger GW Pryor RW: Effects of filter pore size on efficacy of continuous arteriovenous hemofiltration therapy for Staphylococcus aureus-induced septicemia in immature swine. *Crit Care Med.* 1998;26(4):730–737. 9559612 10.1097/00003246-199804000-00024

[54] Morgera S Haase M Kuss T: Pilot study on the effects of high cutoff hemofiltration on the need for norepinephrine in septic patients with acute renal failure. *Crit Care Med.* 2006;34(8):2099–2104. 10.1097/01.CCM.0000229147.50592.F9 16763508

[55] Peng ZY Kiss JE Cortese-Hasset A: Plasma filtration on mediators of thrombotic microangiopathy: an *in vitro* study. *Int J Artif Organs.* 2007;30(5):401–406. 17551903 10.1177/039139880703000507

[56] Formica M Inguaggiato P Bainotti S: Coupled plasma filtration adsorption. *Contrib Nephrol.* 2007;156:405–410. 17464151 10.1159/000102131

[57] Bellomo R Tetta C Ronco C: Coupled plasma filtration adsorption. *Intensive Care Med.* 2003;29(8):1222–1228. 10.1007/s00134-003-1796-x 12830374

[58] Ronco C Brendolan A Lonnemann G: A pilot study of coupled plasma filtration with adsorption in septic shock. *Crit Care Med.* 2002;30(6):1250–1255. 10.1097/00003246-200206000-00015 12072677

[59] Ronco C Ricci Z De Backer D: Renal replacement therapy in acute kidney injury: controversy and consensus. *Crit Care.* 2015;19:146. 10.1186/s13054-015-0850-8 25887923 PMC4386097

[60] Ronco C: The Charta of Vicenza. *Blood Purif.* 2015;40(1):I–V. 26201815 10.1159/000437399

[61] Clark E Molnar AO Joannes-Boyau O: High-volume hemofiltration for septic acute kidney injury: a systematic review and meta-analysis. *Crit Care.* 2014;18(1):R7. 10.1186/cc13184 24398168 PMC4057068

[62] Livigni S Bertolini G Rossi C: Efficacy of coupled plasma filtration adsorption (CPFA) in patients with septic shock: a multicenter randomised controlled clinical trial. *BMJ Open.* 2014;4(1):e003536. 10.1136/bmjopen-2013-003536 24401721 PMC3902195

